# Bis-Benzylisoquinoline Alkaloids Inhibit Porcine Epidemic Diarrhea Virus by Disrupting Virus Entry

**DOI:** 10.3390/pathogens12060845

**Published:** 2023-06-19

**Authors:** Caisheng Zhang, Huan Chen, Liumei Sun, Pu Zhao, Chuanxiang Qi, Ying Yang, Anqi Si, Yingjuan Qian, Yong-Sam Jung

**Affiliations:** 1 One Health Laboratory, Jiangsu Foreign Expert Workshop, College of Veterinary Medicine, Nanjing Agricultural University, Nanjing 210095, China; 2 MOE Joint International Research Laboratory of Animal Health and Food Safety, College of Veterinary Medicine, Nanjing Agricultural University, Nanjing 210095, China; 3 Jiangsu Key Laboratory of Sericultural Biology and Biotechnology, School of Biotechnology, Jiangsu University of Science and Technology, Zhenjiang 212100, China; 4 Jiangsu Key Laboratory for High-Tech Research and Development of Veterinary Biopharmaceuticals, Jiangsu Agri-Animal Husbandry Vocational College, Veterinary Bio-Pharmaceutical, Taizhou 225300, China

**Keywords:** berbamine, fangchinoline, (+)-fangchinoline, PEDV entry, lysosome acidification

## Abstract

The porcine epidemic diarrhea virus (PEDV), belonging to the α-coronavirus, is the causative agent of porcine epidemic diarrhea (PED). Presently, protection from the existing PEDV vaccine is not effective. Therefore, anti-PEDV compounds should be studied. Berbamine (BBM), Fangchinoline (FAN), and (+)-Fangchinoline (+FAN), are types of bis-benzylisoquinoline alkaloids that are extracted from natural medicinal plants. These bis-benzylisoquinoline alkaloids have various biological activities, including antiviral, anticancer, and anti-inflammatory properties. In this study, we found that BBM, FAN, and +FAN suppressed PEDV activity with a 50% inhibitory concentration of 9.00 µM, 3.54 µM, and 4.68 µM, respectively. Furthermore, these alkaloids can decrease the PEDV-N protein levels and virus titers in vitro. The time-of-addition assay results showed that these alkaloids mainly inhibit PEDV entry. We also found that the inhibitory effects of BBM, FAN, and +FAN on PEDV rely on decreasing the activity of Cathepsin L (CTSL) and Cathepsin B (CTSB) by suppressing lysosome acidification. Taken together, these results indicated that BBM, FAN, and +FAN were effective anti-PEDV natural products that prevented PEDV entry and may be considered novel antiviral drugs.

## 1. Introduction

The porcine epidemic diarrhea virus (PEDV) belongs to the α-coronavirus family and can infect pigs of all ages, causing acute diarrhea, vomiting, dehydration, and high mortality in neonatal piglets. PED causes large-scale outbreaks and is one of the most important diseases that harm the pig industry. This results in significant economic losses worldwide each year, especially after the emergence of highly pathogenic PEDV variant strains [1]. PEDV is an enveloped single-stranded positive-sense RNA coronavirus with a genome of approximately 28 kb. It synthesizes the following four structural proteins spike (S), envelope (E), membrane (M), and nucleocapsid (N), and 17 nonstructural proteins, namely nsp1-nsp16 and ORF3 [2,3].

The PEDV lifecycle comprises the following four stages: binding, entry, replication, and release. PEDV entry into the host cells can be divided into three steps. The PEDV particles first enter cells via endocytosis. Internalized viruses are then trafficked by the endo-lysosome system to accomplish membrane fusion. Finally, the viral genome is released into the cytoplasm [4]. During the entry process, Cathepsin L (CTSL) and Cathepsin B (CTSB), two types of endo-lysosomal enzymes, are required to alter the conformation of the PEDV-S to activate membrane fusion [5,6]. Some previous studies have indicated that the maximal activity of CTSL and CTSB requires endo-lysosomes to have a highly acidic pH [7,8]. Therefore, PEDV entry requires an acidic environment.

Owing to the difference in antigens, namely the genes (>10% amino acid variation between respective S proteins) and phylogeny among the vaccine strains and prevalent strains of the PEDV (G1 versus G2), the existing vaccines are not effective in preventing and controlling PEDV [9,10,11]. Broad-spectrum antiviral compounds that target host cells are possibly toxic [12]. Therefore, studying low-toxic antiviral compounds is a key route for the prevention and control of PEDV. Berbamine (BBM), Fangchinoline (FAN), and (+)-Fangchinoline (+FAN) are a few types of bis-benzylisoquinoline alkaloids that are extracted from natural medicinal plants [13]. These three compounds have various biological activities, including antiviral, anticancer, and anti-inflammatory activities, by modulating different cell-signaling pathways [14,15,16]. In a study of antiviral drugs, BBM was reported to inhibit Middle Respiratory Syndrome Coronavirus (MERS-CoV), Severe Acute Respiratory Syndrome Coronavirus 2 (SARS-CoV-2), Japanese encephalitis Virus (JEV), Ebola virus (EBOV), and African Swine Fever Virus (ASFV) infections [17,18,19,20,21]. FAN can inhibit MERS-CoV, Human Coronavirus OC43, and human immunodeficiency virus type 1 (HIV-1) [18,22,23]. Mechanistically, BBM and FAN blocked TPC activity, which is required for endosomal motility and MERS pseudovirus translocation through acidic Ca^2+^ stores. Further study showed that BBM prevents SARS-CoV-2 or MERS-CoV by compromising TRPMLs-mediated endolysosomal trafficking of ACE2 or DPP4, the primary receptors of SARS-CoV-2 and MERS-CoV.

In this study, we investigated whether BBM, FAN, and +FAN have anti-PEDV activity. We found that BBM, FAN, and +FAN can suppress PEDV by decreasing the activity of CTSL and CTSB through the suppression of lysosome acidification, thereby inhibiting PEDV entry. Our results indicate the mechanism underlying the inhibitory effects of BBM, FAN, and +FAN on PEDV entry and provide a reference for developing new broad-spectrum antiviral drugs against PEDV.

## 2. Materials and Methods

### 2.1. Cell Culture

The Vero-E6 (African green monkey kidney cells), Marc-145 (a clone of the African monkey kidney MA-104 cell line), PK15 (pig kidney epithelial morphology cells), and A549 (human lung carcinoma epithelial cell line) cells were cultured in Dulbecco’s modified Eagle’s medium (DMEM) (Gibco, NY, USA) supplemented with 8% fetal bovine serum (FBS) (Gibco, NY, USA) and 1% Penicillin/Streptomycin (*w*/*v*) (p/s) in a humidified 5% CO_2_ incubator at 37 °C.

### 2.2. Antibody and Reagents

A rabbit anti-PEDV-N protein polyclonal antibody was generated in the laboratory [24]. A beta-actin rabbit polyclonal antibody was purchased from Proteintech Group, Inc. (Rosemont, IL, USA). Alexa 488-conjugated goat anti-rabbit IgG antibody and 4’,6-diamidino-2-phenylindole (DAPI) were obtained from Thermo Fisher Scientific, Inc. (San Hose, CA, USA). The Cell Counting Kit-8 (CCK-8) was purchased from Apex Bio Technology, Inc. (Houston, TX, USA). A soluble TMB Substrate Solution (TMB) was obtained from Tiangen (Beijing, China). HiScript II Reverse Transcriptase and 2×Taq Master Mix were purchased from Vazyme Biotech Co., Ltd. (Nanjing, China). An RNA extraction kit was obtained from PuDi Biotech Co., Ltd. (Shanghai, China). LysoSensor™ Yellow/Blue DND-160 was purchased from Thermo Fisher Scientific, Inc. (Waltham, MA, USA). Magic Red^®^ Cathepsin L Assay Kit and Magic Red^®^ Cathepsin B Assay Kit were obtained from Immunochemistry Technologies, LLC. (Davis, CA, USA). BBM, FAN, and +FAN were purchased from Selleck Chemicals (Houston, TX, USA). NH4Cl was obtained from Sigma-Aldrich (St. Louis, MO, USA).

### 2.3. PEDV Propagation

The PEDVs (strains HLJBY and CV777) were propagated in Vero-E6 cells. The Vero-E6 cells were infected with PEDV at a multiplicity of infection (MOI) of 0.01 at 37 °C for 1 h. After incubation, the cells were washed with phosphate-buffered saline (PBS) and transferred to DMEM containing 2% FBS and 17.5 ng/mL trypsin. The virus titer was determined by performing the plaque formation assay.

### 2.4. Cytotoxicity Assay

The cytotoxic effects of BBM, FAN, and +FAN on Vero-E6, Marc-145, A549, and PK-15 cells was measured using the CCK-8 assay according to the manufacturer’s protocol. Mechanistically, WST-8 will be reduced by dehydrogenases, which are abundant in viable cells and transformed to formazan, which is an orange-colored dye soluble in the culture medium. The quantity of the formazan generated by dehydrogenases is directly proportional to the number of living cells. Briefly, cells were seeded at 1.5 × 10^4^ per well in a 96-well plate. After culturing for 12 h, the cells were treated with different concentrations of the alkaloid compounds (0, 5, 10, and 20 µM of BBM, FAN, and +FAN). After 14 h, the fresh DMEM containing 10% CCK-8 reagent was added to each well for 2 h, and then the absorbance was measured at 450 nm (BioTek Instruments, Inc, Winoonski, VT, USA). To determine the cell viability against BBM, FAN, and +FAN treatment during PEDV infection, Vero-E6 or Marc-145 cells were seeded at 1.5 × 10^4^ per well in a 96-well plate. After 12 h, the cells were treated with different concentrations of the alkaloid compounds (1, 2, 4, 8 μM of FAN and +FAN, 1, 2, 4, 8, 12, 16 μM of BBM) for 2 h and then infected with 1 MOI PEDV for 1 h at 37 °C. After incubation, the cells were washed with PBS and transferred to DMEM containing 2% FBS and 17.5 ng/mL trypsin with compound treatments. After another 11 h, the fresh DMEM containing 10% CCK-8 reagent was added to each well for 2 h, and then the absorbance was measured at 450 nm.

### 2.5. Determination of the Half Maximal Inhibitory Concentration (IC_50_) of Compounds

The cell-based enzyme-linked immunoassay (ELISA) was performed to determine the half-maximal inhibitory concentration (IC_50_) of the compounds. The Vero-E6 cells were seeded at 1.5 × 10^4^ per well in a 96-well plate. After culturing for 12 h, the cells were treated with different concentrations of the compounds (1, 2, 4, 8 μM of FAN and +FAN, 1, 2, 4, 8, 12, 16 μM of BBM) for 2 h and then infected with 1 MOI PEDV for 1 h at 37 °C. After incubation, the cells were washed with PBS and transferred to DMEM containing 2% FBS and 17.5 ng/mL trypsin with compound treatments. The cells were then fixed and permeabilized using 4% formaldehyde and 0.1% Triton X-100, respectively, at 37 °C for 30 min, washed with Glycine-PBS (0.02 M glycine in PBS), and finally blocked with 3% bovine serum albumin (BSA) in PBS at 37 °C for 1 h. Subsequently, the cells were incubated with the first and secondary antibodies, respectively. Finally, 100 µL of TMB was added, and the cells were incubated at room temperature for 30 min. Subsequently, 50 μL/well of 2% H_2_SO_4_ was dispensed in each well to stop the reaction, and OD_450_ value was measured. OriginPro^®^ 2022 software was utilized to get the IC_50_ value.

### 2.6. The Effect of the Compounds on PEDV Binding and Entry

To investigate the ability of the compounds in inhibiting PEDV binding, a previously described binding assay was modified [25]. Vero-E6 cells were pretreated with the compounds for 2 h (1 h at 37 °C and another 1 h at 4 °C), and then washed with cold PBS and incubated with 1 MOI PEDV, the HLJBY strain, with or without the compound for 1 h at 4 °C. After incubation, the cells were washed thrice with cold PBS to remove the unattached virus. Then, total RNA was extracted to perform reverse transcription–polymerase chain reaction (RT-PCR) to measure the levels of PEDV-M and actin mRNA. For the entry assay, Vero-E6 cells were cooled for 1 h at 4 °C and then infected with 1 MOI PEDV, the HLJBY strain, for 1 h at 4 °C. After infection, they were treated with the compound for 2 h at 37 °C, and then the cells were washed with citrate buffer (pH = 3) to remove non-internalized virions. The cells were then incubated with a fresh culture medium for 4 h. Finally, the cells were harvested to perform the immunoblotting assay and detect the changes in PEDV-N and actin protein.

### 2.7. RNA Extraction and RT-PCR

The total RNA was extracted from the cells using the Simply P Total RNA Extraction Kit (BioFlux, Beijing, China) according to the manufacturer’s protocol. The cDNA was synthesized using the iScript™ Reverse Transcription Supermix kit (Bio-Rad, Hercules, CA, USA) as per the manufacturer’s instructions. The RT-PCR primers were designed using the Primer Premier 5 software. The sequences of the primers used for viral and cellular gene amplification were as follows: PEDV-M-F (5′-GGA CAC ATT CTT GGT GGT CTT TC-3′) and PEDV-M-R (5′-GCC AGT AGC AAC CTT ATA GCC C-3′), actin-F (5′-ACA CTG TGC CCA TCT ACG AGG-3′) and actin-R (5′-TTG CCA ATG GTG ATG ACC TG-3′).

### 2.8. Western Blot Analysis

To detect the N protein levels in PEDV-infected Vero-E6 cells, the cells were lysed in a sodium dodecyl sulfate (SDS) sample buffer (0.1 M Tris-Cl pH 6.8, 20% Glycerol, 4% SDS, 4% β-Mercaptoethanol, 1% Bromophenol Blue). The protein samples were denatured for 10 min at 98 °C, and then separated using an SDS-PAGE gel, and transferred to a polyvinylidene fluoride (PVDF) membrane (Pall Corp, Beijing, China). The membranes were blocked using 3% skim milk at room temperature for 40 min, and then incubated with the primary antibodies overnight at 4 °C, washed thrice with PBST (PBS with Tween 20), and finally incubated with secondary antibodies for 4–6 h at 4 °C. The target protein was detected using the enhanced chemiluminescence (ECL) reagent.

### 2.9. Immunofluorescence Analysis

The Vero-E6 cells seeded on coverslips were fixed and permeabilized using 4% formaldehyde and 0.1% Triton X-100 at 37 °C for 30 min, which was washed with glycine-PBS (0.02 M glycine in PBS) and blocked with 3% BSA in PBS 37 °C for 30 min. They were incubated with the primary antibodies for 1 h at 37 °C, washed thrice with PBS, and then incubated with secondary antibodies for 30 min at 37 °C. After washing thrice with PBS, the nuclei were stained with 4′,6-diamidino-2-phenylindole (DAPI) containing the anti-fade Dabco solution. The images were obtained using a Nikon fluorescence microscope (Nikon Eclipse Ti-U, Tokyo, Japan).

### 2.10. Plaque Formation Assay (PFU)

The Vero-E6 cells were seeded in a 6-well plate. The cells grew to a monolayer after culturing for 12 h. The cells were then infected with the virus and diluted in DMEM through a ten-fold serial dilution for 1 h at 37 °C. Then, a 2 mL overlay medium (1% low-melting agarose, 2% FBS, and 17.5 ng/mL trypsin in DMEM) was added to each well, and the plates were incubated at 37 °C for 2–3 days until the plaques were visible. Then, 500 μL of 1% crystal violet was added and stained for 3–5 h at room temperature. After incubation, the overlay medium with crystal was removed, and the number of plaques was counted.

### 2.11. Lysosomal pH Measurement

The lysosomal pH was determined using lysosensor^TM^ yellow/blue DND-160 reagent. The Vero-E6 cells were seeded at 1.5 × 10^4^/well in a black 96-well plate with a clear bottom. After culture for 12 h, the cells were treated with compounds for 0, 1, 2, and 4 h. The cells were treated with 2 mM lysosensor^TM^ yellow/blue DND-160 reagent for 2 min at 37 °C after washing twice with PBS. The cells were washed twice with cold PBS and transferred to DMEM. Finally, the fluorescence intensity was measured at excitation-365 nm/emission-450 nm and excitation-365 nm/emission-510 nm, and the change of the ratio of excitation-365 nm/emission-450 nm to excitation-365 nm/emission-510 nm reflects the change in lysosomal pH.

### 2.12. Determining the Activity of CTSL and CTSB

The Magic Red^®^ Cathepsin-L Assay Kit and Magic Red^®^ Cathepsin-B Assay Kit were used to monitor the intracellular CTSL and CTSB activities. Vero-E6 cells were seeded at 1.5 × 10^4^ per well in a black 96-well plate with clear bottom. After culture for 12 h, the cells were treated with compounds for 0, 1, 2, and 4 h. The cells were then washed with PBS and treated with the Magic Red^®^ Cathepsin-L Assay Kit or Magic Red^®^ Cathepsin-B Assay Kit at 37 °C for 40 min. Finally, the cells were washed using PBS and transferred to DMEM without phenol red. The fluorescence intensity was measured at excitation-592 nm/emission-628 nm.

### 2.13. Statistical Analysis

All experiments were performed in triplicate. All results were analyzed using GraphPad Prism and are presented as means ± standard deviations (SDs). The statistical significance was determined using the two-tailed Student unpaired-sample t-test (*, *p* < 0.05; **, *p* < 0.01; ***, *p* < 0.001).

## 3. Results

### 3.1. The 50% Cytotoxicity Concentration (CC_50_) and IC_50_ Values of BBM, FAN, and +FAN in Vero-E6 Cells

**Figure 1:**
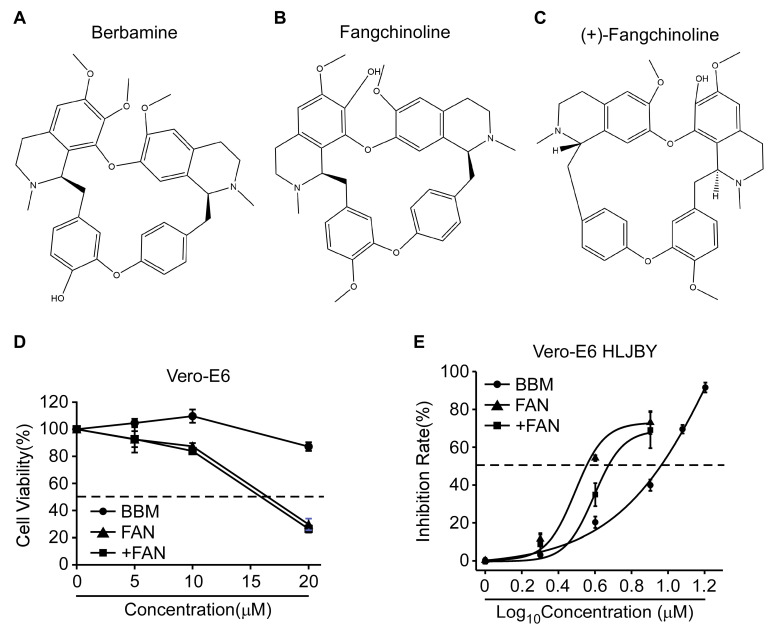
CC_50_ and IC_50_ of Berbamine (BBM), Fangchinoline (FAN), and (+)-Fangchinoline (+FAN). (**A**–**C**) The chemical structures of BBM, FAN, and +FAN. (**D**) Vero-E6 cells were treated with 0, 5, 10, and 20 μM of BBM, FAN, and +FAN for 14 h, and then treated with CCK-8 for 2 h. The OD_450_ value was measured. (**E**) The Half Maximal Inhibitory Concentration (IC_50_) was determined by cell-based ELISA assay and calculated by nonlinear regression analysis. The horizontal dotted lines show 50% cell viability or 50% inhibition rate.

Bis-benzylisoquinoline alkaloids exhibit high anti-viral activity against viruses, especially coronaviruses [26]. BBM, FAN, and +FAN have the same chemical formula and a similar chemical structure (Figure 1A–C). Therefore, we investigated whether all three exhibit anti-PEDV activity. First, we determined the cytotoxic effects of BBM, FAN, and +FAN on Vero-E6 cells by performing the CCK-8 assay. The CC_50_ values of the compounds were more than 20, 17, and 16 µM in the Vero-E6 cells, respectively (Figure 1D). Then, the inhibitory effects of BBM, FAN, and +FAN against PEDV, HLJBY strain infection, were analyzed by the cell-based ELISA. The IC_50_ values of BBM, FAN, and +FAN were 9.00, 3.54, and 4.68 μM, respectively (Figure 1E). Further, the selectivity indices (CC_50_/IC_50_) of BBM, FAN, and +FAN were >2.22, 4.8, and 3.41, respectively (Table 1). Simultaneously, we determined the CC_50_ values of these compounds in Marc-145 cells and the IC_50_ values against PEDV, HLJBY strain, in Marc-145 cells (Figure S1A,B). Furthermore, we detected the IC_50_ values of BBM, FAN, and +FAN against the CV777 strain in Vero-E6 and Marc-145 cells (Figure S1C,D). Meanwhile, the cytotoxic effect of Vero-E6 or Marc-145 cells against PEDV, CV777 or HLJBY strain, infection (one MOI) with BBM, FAN, and + FAN treatments were determined (Figure S1 E–H). The cell viability all remained above 90% upon PEDV infection with the compounds’ treatment. Generally, these data showed that an effective dose of BBM, FAN, and +FAN exhibited a certain degree of anti-PEDV activity. Finally, we selected a compound concentration (BBM = 10 μM, FAN and +FAN = 5 μM) with low cytotoxicity and an obvious effect on the Vero-E6 cells for further study.

### 3.2. PEDV Infection Was Inhibited by BBM, FAN, and +FAN In Vitro

**Figure 2:**
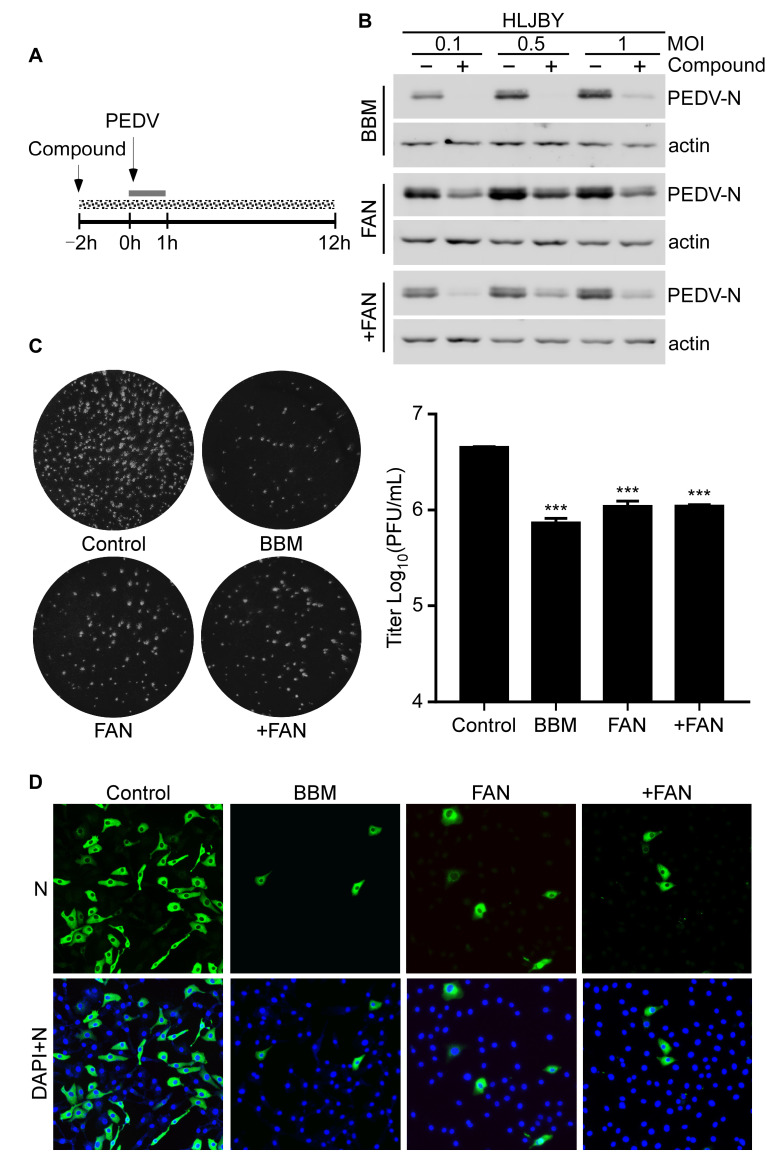
BBM, FAN, and +FAN inhibit PEDV infection in vitro. (**A**) Overall scheme for Vero-E6 cells infection and treatment with BBM (10 μM), FAN (5 μM), and +FAN (5 μM); the solid gray line refers to the PEDV infection period and the dotted gray line refers to the compound treatment. (**B**) The protein sample was harvested at 12 h post-infection (h.p.i.) and PEDV-N and actin protein levels were determined by Western blotting. (**C**) The cells were harvested at 12 h.p.i., and the PEDV, HLJBY strain titer was determined by the PFU assay. (**D**) The cells were fixed and permeabilized at 12 h.p.i. and PEDV-N fluorescence intensity was determined by an IFA assay. The results were analyzed using the Student *t*-test (***, *p* < 0.001).

To determine the antiviral activities of BBM, FAN, and +FAN, Vero-E6 cells were infected with one MOI PEDV and treated with these compounds (Figure 2A). The solid gray line in Figure 2A refers to the PEDV infection period, and the dotted gray line refers to BBM, FAN, and +FAN treatments. HLJBY-N protein levels decreased markedly after BBM, FAN, and +FAN treatments in Vero-E6 and Marc-145 cells (Figure 2B and Figure S2A). Similarly, the CV777-N protein was dramatically decreased by BBM, FAN, and +FAN treatments in Vero-E6 and Marc-145 cells (Figure S2B,C). Furthermore, PEDV titers decreased markedly by BBM, FAN, and +FAN treatments (Figure 2C). Additionally, the inhibitory effects of BBM, FAN, and +FAN on PEDV replication were determined by immunofluorescence staining. We observed that the PEDV-N fluorescence intensity decreased after BBM, FAN, and +FAN treatments (Figure 2D). These data suggested that BBM, FAN, and +FAN could markedly inhibit PEDV infection in vitro.

### 3.3. BBM, FAN, and +FAN Inhibited PEDV Infection Mainly at the Early Stage

**Figure 3:**
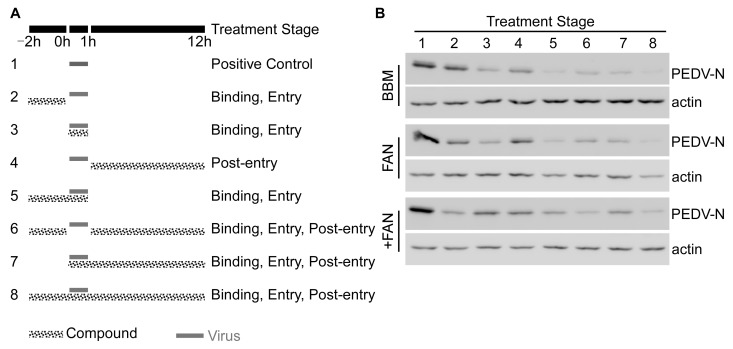
Time-of-addition analysis of BBM, FAN, and +FAN. (**A**) Overall scheme for Vero-E6 cells infection and treatment with BBM (10 μM), FAN (5 μM), and +FAN (5 μM); the solid gray line refers to the PEDV infection period and the dotted gray line refers to the BBM, FAN, and +FAN treatment. (**B**) After 12 h.p.i., the protein sample was harvested, and the PEDV-N and actin protein levels were determined by Western blotting.

The PEDV lifecycle comprises four stages: binding, entry, replication, and release. To determine which stage of the virus life cycle was affected by BBM, FAN, and +FAN, we performed a time-of-drug-addition assay. The compounds were added at three stages: pre-treatment, co-treatment, or post-infection (Figure 3A). The results showed that PEDV-N protein levels decreased after BBM, FAN, and +FAN addition at all stages. PEDV-N protein level decreased most significantly after BBM and FAN addition at the co-treatment stage and +FAN addition at the pre-treatment stage (Figure 3B). Therefore, BBM, FAN, and +FAN mainly inhibit PEDV infection at the early stage.

### 3.4. BBM, FAN, and +FAN Did Not Target PEDV Particles Directly

**Figure 4:**
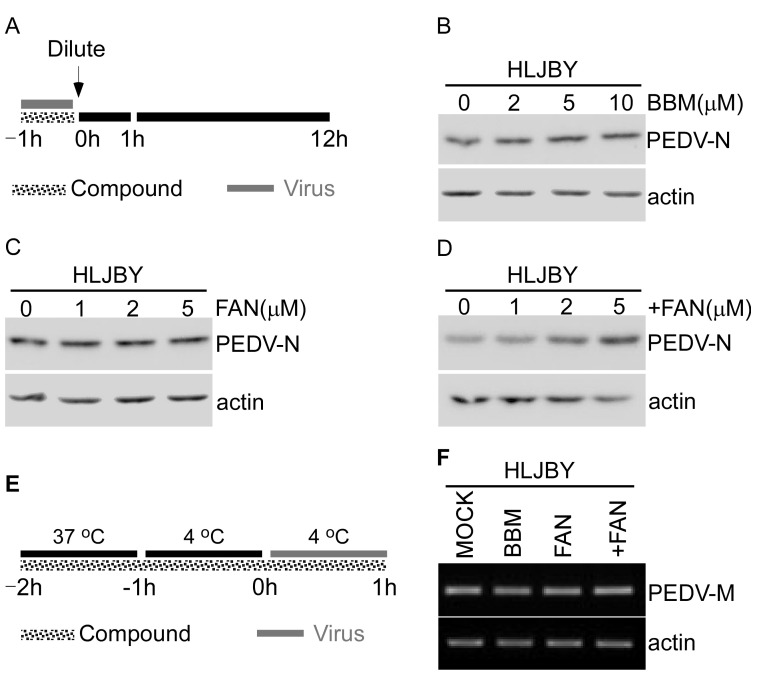
BBM, FAN, and +FAN did not target PEDV particles directly. (**A**) The overall scheme for Vero-E6 cells infection and treatment with BBM (0, 2, 5, and 10 μM), FAN (0, 1, 2, and 5 μM), and +FAN (0, 1, 2, and 5 μM). PEDV, HLJBY strain (one MOI) was mixed with BBM, FAN, and +FAN for 1 h at 37 °C. Then, the mixture was diluted in 2 mL DMEM and incubated with Vero-E6. (**B**–**D**) The protein sample was harvested at 12 h.p.i., and the PEDV-N and actin protein levels were determined by Western blotting. (**E**) An overall scheme for PEDV infection and treatment with BBM (10 μM), FAN (5 μM), and +FAN (5 μM). The gray solid line refers to the PEDV infection period. The dotted gray line refers to the BBM, FAN, and +FAN treatments. (**F**) Vero-E6 cells were pretreated with BBM, FAN, and +FAN for 2 h (1 h at 37 °C and another at 4 °C), infected with PEDV, HLJBY strain (one MOI), and then treated with BBM, FAN, and +FAN for 1 h at 4 °C. Finally, the cells were washed with cold PBS to wash out the unbound virus, followed by RT-PCR assay to determine the levels of PEDV-M and actin mRNA.

The study of the mechanism of antiviral compounds is focused on two different points: whether such compounds target corresponding viruses directly or indirectly via host cell factors [27]. Therefore, we determined whether BBM, FAN, and +FAN exerted a direct effect on virions. To assess this, we premixed PEDV with BBM, FAN, and +FAN for 1 h at 37 °C, and then diluted the mixture before inoculating the cells (Figure 4A). The Western blotting results showed that PEDV-N protein levels did not change much after BBM, FAN, and +FAN or dimethyl sulfoxide (DMSO) treatments (Figure 4B–D). The results suggested that BBM, FAN, and +FAN did not target PEDV particles. To investigate the antiviral activity of BBM, FAN, and +FAN, we investigated at which stage of the PEDV early infection process (viral binding or entry) these compounds were most effective. Specifically, we incubated Vero-E6 cells at 37 °C for 1 h and at 4 °C for 1 h to precool and then infected them with PEDV at 4 °C. The Vero-E6 cells were treated with the three compounds throughout the process (Figure 4E). We found that the mRNA levels of PEDV-M did not differ in cells treated with or without the compounds (Figure 4F). These data indicated that BBM, FAN, and +FAN did not exert their effects on PEDV at the adsorption stage.

### 3.5. BBM, FAN, and +FAN Inhibited PEDV Entry by Suppressing Lysosome Acidification

**Figure 5:**
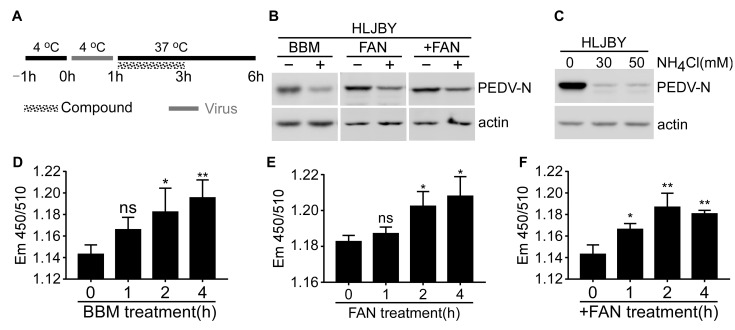
BBM, FAN, and +FAN inhibited PEDV entry by suppressing endo-lysosome acidification. (**A**) The overall scheme for PEDV infection and treatment with BBM (10 μM), FAN (5 μM), and +FAN (5 μM). The gray solid line refers to the PEDV infection period. The dotted gray line refers to the BBM, FAN, and +FAN treatments. (**B**) Vero-E6 cells were infected with PEDV, HLJBY strain (one MOI) at 4 °C. After infection, the cells were treated with BBM, FAN, and +FAN for 2 h at 37 °C and washed thrice with citrate buffer, followed by incubation in fresh DMEM for 3 h. Finally, the protein sample was harvested, and the levels of PEDV-N and actin protein were determined by Western blotting. (**C**) Vero-E6 cells were infected with PEDV, HLJBY strain (one MOI) at 4 °C. After infection, the cells were treated with NH4Cl (0, 30, and 50 mM) for 2 h at 37 °C and washed thrice with citrate buffer, followed by incubation with fresh DMEM for 3 h. Finally, the protein sample was harvested, and the levels of PEDV-N and actin protein were determined by Western blotting. (**D**–**F**) The Vero-E6 cells were treated with BBM, FAN, and +FAN for 0, 1, 2, and 4 h, and then labeled with 2 μM lysosensor^TM^ yellow/blue DND-160 for 3 min at 37 °C. The excess dye was removed with cold PBS and then transferred to DMEM. The emission ratio at 451/510 nm was measured at excitation-365 nm. The results were analyzed by Student t-test (*, *p* < 0.05; **, *p* < 0.01; ns, *p* > 0.05).

Our data showed that BBM, FAN, and +FAN inhibited PEDV infection mainly at the early stage and hardly affected viral binding to cells. Therefore, whether BBM, FAN, and +FAN inhibited PEDV during the entry stage was investigated in the present study. The PEDV entry assays were performed as shown in Figure 5A. PEDV virions were bound to the cell membrane of Vero-E6 cells at 4 °C, then fresh DMEM, with or without BBM, FAN, and +FAN, was added at 37 °C for 2 h. Next, non-internalized PEDV virions were removed using the citrate buffer. The Western blotting results revealed that the PEDV-N protein levels decreased after BBM, FAN, and +FAN treatments compared with that after DMSO treatment (Figure 5B). These results suggested that BBM, FAN, and +FAN exerted an antiviral effect during the viral entry stage.

Ammonium chloride is a weak base that increases the pH of acidic cell compartments [28]. A previous study showed that ammonium chloride inhibited PEDV entry into cells by inhibiting lysosome acidification [4]. We observed that the HLJBY-N protein level decreased after treatment with 30 mM and 50 mM ammonium chloride during the viral entry stage (Figure 5C). These data showed that the entry of the PEDV, HLJBY strain, in the cells depended on lysosome acidification. Additionally, previous results showed that BBM, FAN, and +FAN inhibited PEDV entry into cells. Therefore, we investigated whether BBM, FAN, and +FAN affected lysosome acidification. The Lysosensor^TM^ yellow/blue DND-160 was used to measure the pH of the lysosome. The results showed that the pH of the lysosome increased after BBM, FAN, and +FAN treatments (Figure 5D–F). These data indicated that BBM, FAN, and +FAN inhibited PEDV entry by suppressing lysosome acidification.

### 3.6. BBM, FAN, and +FAN Decreased the Activity of CTSL and CTSB

**Figure 6:**
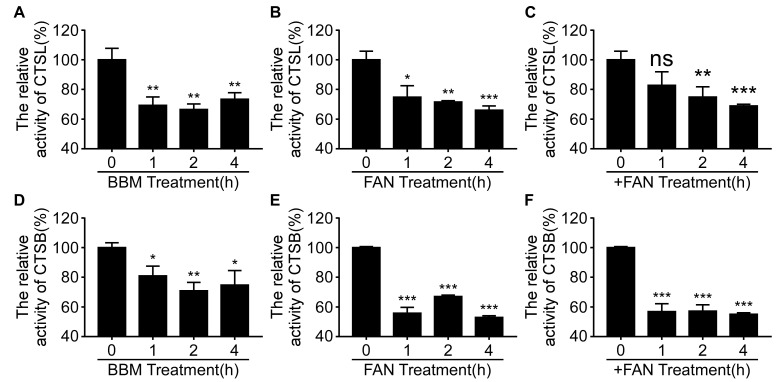
BBM, FAN, and +FAN inhibit PEDV entry by decreasing the activity of CTSL and CTSB. (**A**–**C**) The Vero-E6 cells were treated with BBM (10 μM), FAN (5 μM), and +FAN (5 μM) for 0, 1, 2, and 4 h and then labeled with the Magic Red^®^ Cathepsin-L Assay Kit at 37 °C for 40 min. The excess dye was removed with PBS, followed by transferring it to DMEM without phenol red. The fluorescence intensity was measured at excitation-592 nm/emission-628 nm. (**D**–**F**) The Vero-E6 cells were treated with BBM (10 μM), FAN (5 μM), and +FAN (5 μM) for 0, 1, 2, and 4 h, followed by labeled with the Magic Red^®^ Cathepsin-B Assay Kit at 37 °C for 40 min. The excess dye was removed with PBS, followed by transferring it to DMEM without phenol red. The fluorescence intensity was measured at excitation-592 nm/emission-628 nm. The results were analyzed by Student t-test (*, *p* < 0.05; **, *p* < 0.01; ***, *p* < 0.001).

The S protein of PEDV was activated by endo-lysosomal cysteine proteases, such as CTSL and CTSB, to expose a fusion domain for membrane fusion and viral genome translocation to the cytoplasm [5,6]. CTSL and CTSB require low pH for their optimal activity. As shown in Figure 5D–F, the lysosomal pH increased after BBM, FAN, and +FAN treatment. Therefore, we investigated whether BBM, FAN, and +FAN decreased the activity of the CTSL and CTSB using the Magic Red^®^ Cathepsin-L Assay Kit and Magic Red^®^ Cathepsin-B Assay Kit. We found that the fluorescence intensities decreased after BBM, FAN, and +FAN treatments, indicating decreased activity of CTSL and CTSB (Figure 6A–F). The data indicated that BBM, FAN, and +FAN decreased the activity of CTSL and CTSB.

**Figure 7:**
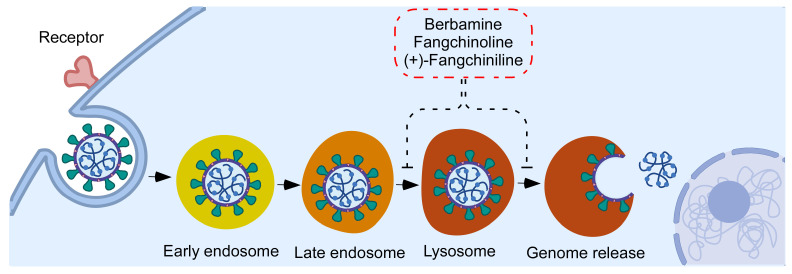
Diagram showing a hypothesized antiviral mechanism of BBM, FAN, and +FAN during PEDV infection.

The endo-lysosome system and CTSL and CTSB are required to release the PEDV genome from the internalized viral particles. As illustrated in Figure 7, BBM, FAN, and +FAN inhibited PEDV entry by suppressing the endo-lysosome traffic and the activity of CTSL and CTSB by increasing the pH of the lysosome. Further, the low activity of CTSL and CTSB could not cleave the PEDV-S protein to expose the fusion domain, thus suppressing membrane fusion. Ultimately, the viral genome could not translocate to the cytoplasm.

## 4. Discussion

PED is a highly contagious disease in pigs, causing a significant economic loss in the swine industry [29]. The hypervariability of the PEDV has led to complex and heterogeneous field pandemics, and efficient vaccines are difficult to develop [30]. Therefore, developing an efficient antiviral compound against PEDV infection is crucial. BBM, FAN, and +FAN are bis-benzylisoquinoline alkaloids with anticancer, anti-inflammatory, and antiviral activities [26,31,32]. Here, we have found that they exerted anti-PEDV activities, revealing some of their functional mechanisms. To investigate the anti-PEDV mechanism of the compounds in vitro, we performed the time-of-drug-addition assay. The results showed that BBM and FAN exerted better anti-PEDV activity during the co-treatment stage, whereas the +FAN was more effective during the pre-treatment stage. We speculate that BBM and FAN share higher similarity in their chemical scaffold structure compared with +FAN, which may lead to +FAN exerting more effective antiviral activity in the pre-treatment stage. In general, these compounds all inhibit PEDV infection at the early stage.

To further investigate which lifecycle stage of PEDV infection was affected by these compounds, we performed PEDV virion binding and entry assays considering the mechanism that the PEDV virion can only bind to the cell membrane but cannot enter the cell at 4 °C. After virus adsorption, virions undergo penetration and uncoating stages before viral gene transcription and replication. In addition, our results showed that these compounds mainly inhibited PEDV entry but not viral attachment. Furthermore, due to some bis-benzylisoquinolines alkaloids regulating endo-lysosome Ca^2+^ release [18], we hypothesize that these compounds inhibit PEDV infection at the viral uncoating stage.

Bis-benzylisoquinolines alkaloids can be divided into two groups: tubocurarine-like compounds and tetrandrine-like compounds. BBM, FAN, and +FAN are tetrandrine-like compounds. According to some study reports, Tetrandrine and Fangchinoline block two-pore channel (TPC) activity, an endo-lysosomal Ca^2+^ channel, suppressing Ca^2+^ release from endo-lysosome [18,33]. Therefore, we speculated that the molecular mechanism by which BBM, FAN, and +FAN inhibit PEDV entry depended on blocking the TPC activity and suppressing the endo-lysosomal Ca^2+^/H^+^ exchanges to suppress the endo-lysosomal acidification. We performed an assay to validate our assumption. The result was consistent with the previous conjecture. CTSL and CTSB, endo-lysosomal proteases, are required for PEDV membrane fusion, a prerequisite for the viral uncoating stage [5]. Suppressed endo-lysosomal acidification decreases CTSL and CTSB activities. Our results showed that BBM, FAN, and +FAN decreased the activity of CTSL and CTSB, furthering PEDV membrane fusion and genome release.

In this study, we found that Berbamine analogs were a novel anti-PEDV drug candidate. Berbamine is a bis-benzylisoquinoline alkaloid isolated from the traditional Chinese herb Berberis amurensis [14]. Berbamine inhibits various viruses, including SARS-CoV-2, MERS-CoV, JEV, ASFV, and EBOV [17,18,19,20,21]. For human coronaviruses, berbamine prevents SARS-CoV-2 or MERS-CoV from entering host cells by decreasing the viral receptor levels of ACE2 or DPP4 at the plasma membrane [17]. For EBOV, berbamine hydrochloride disrupts the EBOV glycoprotein (GPcl) interaction with viral receptor Niemann-Pick C1, thus blocking the fusion of viral and cellular membranes [21]. Moreover, Berbamine prevented JEV entry by blocking endo-lysosomal trafficking [19]. In our study, we found that Berbamine analogs inhibited PEDV infection at the early stage, probably blocking host proteins and subsequently inhibiting virus entry. For compound–protein interactions (CPIs), further analytic tools, such as computational molecular docking analysis, are required to determine the interaction between Berbamine and host proteins. However, PEDV entry/trafficking-associated proteins are not well understood yet.

While studying the inhibition of PEDV entry by BBM, FAN, and +FAN treatments, we found these compounds to be broad-spectrum antiviral compounds. First, our results showed that BBM, FAN, and +FAN inhibited the Influenza A virus (IAV), Herpes simplex virus 1 (HSV-1), and Porcine α-herpesvirus pseudorabies (PRV) infections (Figure S3). Second, diverse viruses, including Coronavirus (SARS-CoV, MERS-CoV, and SARS-CoV-2), Filoviruses (Ebola virus), Orthomyxoviridae (IAV), and Calicivirus (Murine norovirus and Feline calicivirus), entered the host cells with the help of endo-lysosomal cysteine proteases, such as CTSL and/or CTSB [7,34,35,36,37,38,39,40]. Finally, several studies showed that the entry of many viruses, such as Hepatitis B virus (HBV), African swine fever virus (ASFV), and PRV, was acid-dependent [41,42,43,44,45,46,47].

In conclusion, our study shows that natural products such as bis-benzylisoquinoline alkaloids inhibit PEDV at the early stage of infection, exhibiting strong antiviral activitites. Thus, bis-benzylisoquinoline alkaloids may be a potential antiviral drug for PEDV prevention or cure in the future.

## Figures and Tables

**Table 1:** CC_50_, IC_50_, and selectivity indices (CC_50_/IC_50_) of BBM, FAN, and +FAN.

PEDV	Compound	CC_50_ (μM)	IC_50_ (μM)	SI (CC_50_/IC_50_)
HLJBY	BBM	>20	9	>2.22
	FAN	17	3.54	4.8
	+FAN	16	4.68	3.41

## Data Availability

Not applicable.

## Supplementary Materials

The following supporting information can be downloaded at: https://www.mdpi.com/article/10.3390/pathogens12060845/s1, Figure S1: CC50 and IC50 of BBM, FAN, and +FAN; Figure S2: BBM, FAN, and +FAN inhibit PEDV infection in vitro; Figure S3: The CC50 values of BBM, FAN, and +FAN in PK-15 and A549 cells, and the inhibitory effect of the compounds in IAV, HSV, and PRV.
