# Supplementary material for: Bis-Benzylisoquinoline Alkaloids Inhibit Porcine Epidemic Diarrhea Virus by Disrupting Virus Entry
Source: Pathogens. 2023 Jun 19;12(6):845. doi: 10.3390/pathogens12060845 (PMC10304817; doi:10.3390/pathogens12060845)
Supplement: Supplementary file 1 [file pathogens-12-00845-s001.zip › pathogens-2383353-supplementary.pdf]

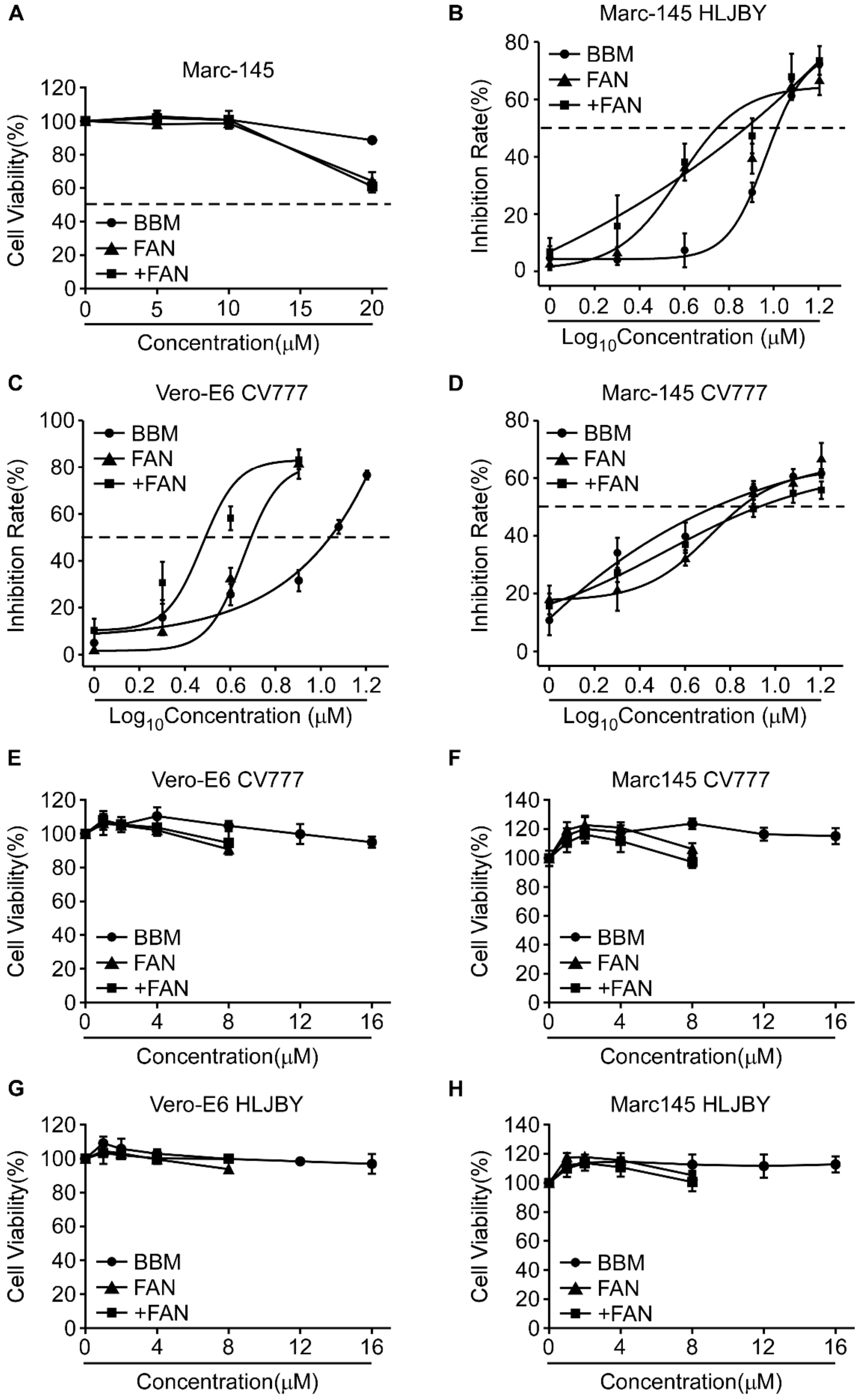

Figure S1. CC_{50} and IC_{50} of BBM, FAN, and +FAN. **(A)** The cytotoxic effect of BBM, FAN, and + FAN was determined in Marc-145. **(B)** The IC_{50} of BBM, FAN, and +FAN was determined in Marc-145 cells. Marc-145 cells were infected with PEDV, HLJBY strain (1 MOI). **(C and D)** The IC_{50} of BBM, FAN, and +FAN was determined in both Vero-E6 and Marc-145 cells. Vero-E6 and Marc-145 cells were infected with PEDV, CV777 strain (1 MOI). **(E and F)** The cytotoxic effect of Vero-E6 or Marc-145 cells against PEDV, CV777 strain infection (1MOI) with various concentrations of BBM, FAN, and + FAN treatments were determined. **(G and H)** The cytotoxic effect of Vero-E6 or Marc-145 cells

against PEDV, HLJBY strain infection (1MOI) with various concentrations of BBM, FAN, and + FAN treatments were determined.

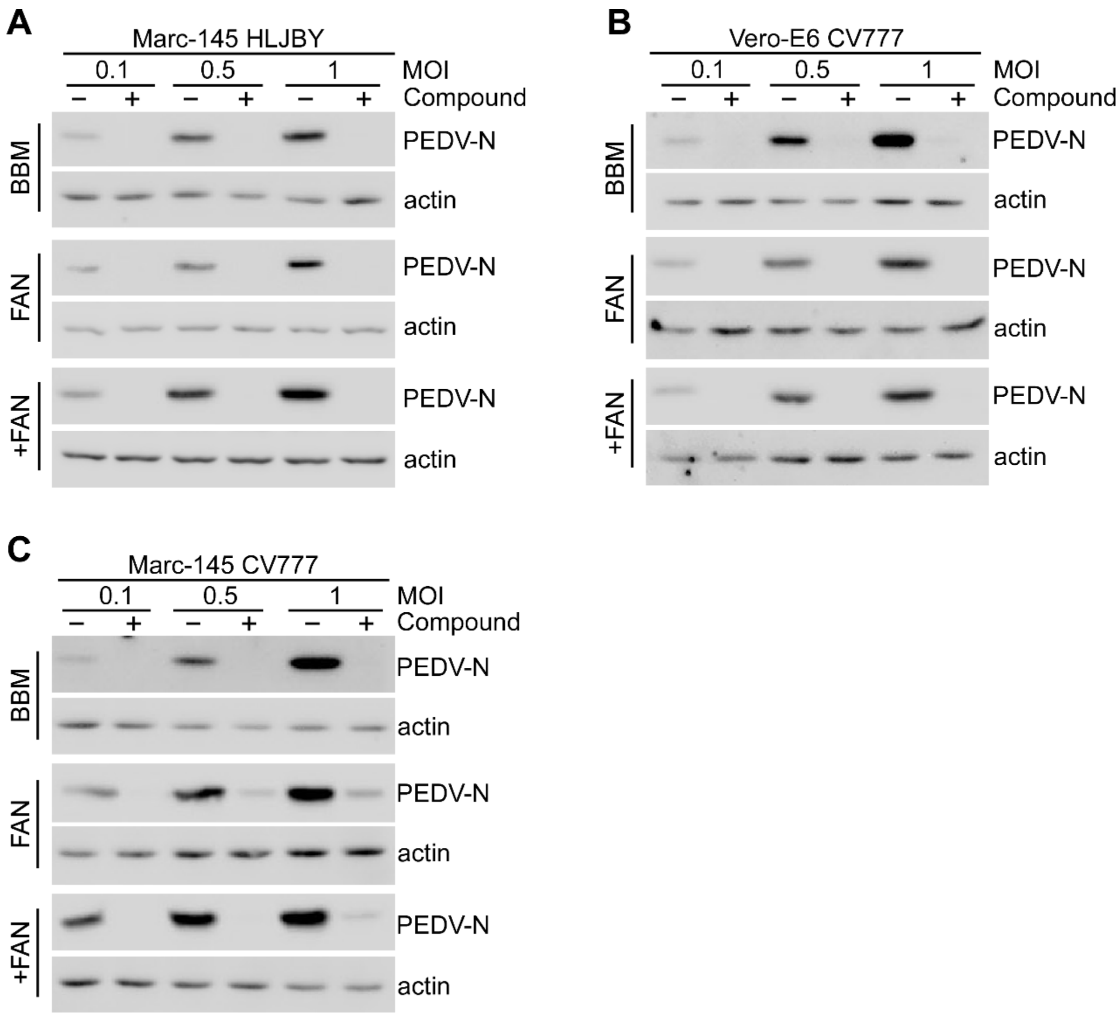

Figure S2. BBM, FAN, and +FAN inhibit PEDV infection *in vitro*. The cells were treated as described in Fig. 2B, the sample were harvested and PEDV-N and actin protein levels were determined using Western Blot assay. **(A)** Marc-145 cells were pretreated with the compounds and then infected with HLJBY. **(B and C)** Vero-E6 and Marc-145 cells were pretreated with the compounds and then infected with CV777.

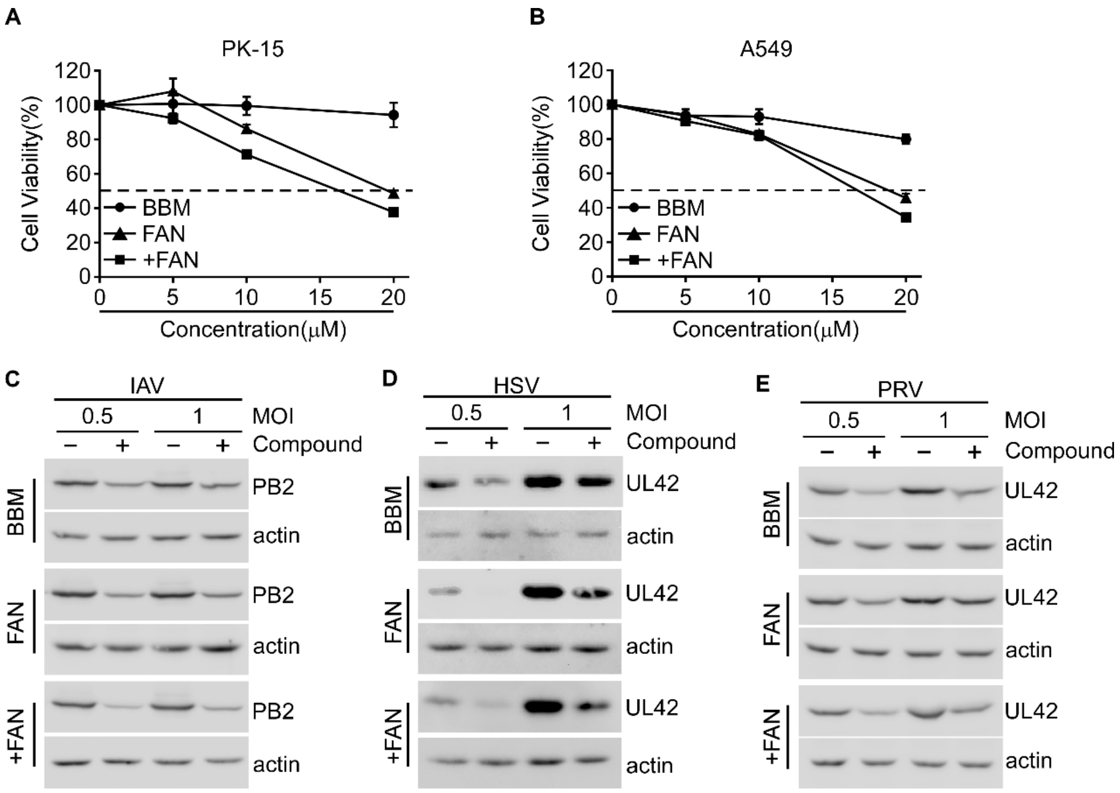

Figure S3. The CC_{50} values of BBM, FAN, and +FAN in PK-15 and A549 cells, and the inhibitory effect of the compounds in IAV, HSV, and PRV. (A and B) The cytotoxic effect of BBM, FAN, and + FAN was determined in PK-15 and A549 cells. (C) A549 cells were pretreated with the indicated compounds and then infected with IAV. (D) Vero-E6 cells were pretreated with the indicated compounds and then infected with HSV. (E) PK-15 cells were pretreated with the indicated compounds and then infected with PRV.
